# Targeting Coping to Improve Surgical Outcomes in Pediatric Patients With Median Arcuate Ligament Syndrome: Feasibility Study

**DOI:** 10.3389/fpsyg.2021.695435

**Published:** 2021-10-22

**Authors:** Colleen Stiles-Shields, Sylwia Osos, Anna Heilbrun, Estée C. H. Feldman, Grace Zee Mak, Christopher L. Skelly, Tina Drossos

**Affiliations:** ^1^Section of Community Behavioral Health, Department of Psychiatry and Behavioral Sciences, Rush University Medical Center, Chicago, IL, United States; ^2^Department of Psychiatry and Behavioral Sciences, The University of Chicago Medicine, Chicago, IL, United States; ^3^College of Arts and Sciences, Roosevelt University, Chicago, IL, United States; ^4^Tandem Psychology Group, Chicago, IL, United States; ^5^Rosalind Franklin University of Medicine and Science, North Chicago, IL, United States; ^6^Section of Pediatric Surgery, Department of Surgery, The University of Chicago Medicine, Chicago, IL, United States; ^7^Section of Vascular Surgery, Department of Surgery, The University of Chicago Medicine, Chicago, IL, United States

**Keywords:** median arcuate ligament syndrome, chronic abdominal pain, pediatric, cognitive behavioral therapy, coping

## Abstract

**Background:** Median arcuate ligament syndrome (MALS) is a vascular compression syndrome leading to postprandial epigastric pain, nausea, and weight loss; it can be treated surgically. While most patients report improved quality of life following surgical intervention, 30% continue to experience chronic abdominal pain. Pre-surgical diagnoses of depression and/or anxiety have been found to significantly predict post-surgical: quality of life, highest experience of pain, anxiety, and parent- and self-reported coping strategies. As such, increasing the coping strategies of pediatric patients with MALS may impact their post-surgical outcomes. The purpose of the current study was to: (1) implement a pre-operative cognitive behavioral therapy protocol with a focus on psychoeducation and coping strategies; and (2) determine feasibility of a pre-surgical intervention for this population.

**Method:** Children (<18 years of age) with a diagnosis of MALS who were eligible for surgical intervention were invited to participate in a 7-week in-person or video-based pre-surgical cognitive behavioral therapy intervention. Psychiatric comorbidities were assessed at baseline and post-surgery; patient-reported distress, pain interference and intensity, health-related quality of life, and health status were assessed at four time points (baseline, week 4, week 7, and post-surgery). Descriptive analyses were used to characterize the sample, assess feasibility outcomes (i.e., attrition rates), and explore symptom-based outcomes across time.

**Results:** Twelve pediatric patients (*M* age = 15.2 ± 1.7; 91.7% female) and their parents (91.7% mothers) participated. Feasibility metrics based on protocol completion were exceeded for engagement at the stages of consent (68.4% vs. goal of ≥50%), treatment initiation (92.3% vs. 85%), and treatment completion (84.6% vs. 75%). Out of the 12 participants, nine (75%) met criteria for at least one comorbid psychiatric diagnosis at baseline and nine (75%) elected to undergo MALS surgery after completing the intervention.

**Conclusion:** The intervention implementation was feasible, despite chronic pain symptoms experienced by the sample, a high prevalence of psychiatric diagnoses, and an international pandemic, suggesting that it would be beneficial to further evaluate the efficacy of the intervention. Future research should include stakeholder input in the design, deployment, and evaluation of a pilot efficacy trial of pre-surgical cognitive behavioral therapy for pediatric patients with MALS.

## Introduction

Chronic abdominal pain (CAP) is a common medical concern among children and adolescents, with 8–20% of youth reporting pain symptomatology so severe that daily functioning is impeded (Perquin et al., 2003). CAP is associated with many negative psychosocial outcomes, including increased risk of depression and anxiety (Campo et al., 2004), decreased health-related quality of life (QOL; Warschburger et al., 2014), diminished perceived social competence (Scharff, 1997; Eccleston et al., 2008), frequent school absences (Ramchandani et al., 2007), diminished school-related functioning (Greco et al., 2007), and higher levels of peer victimization (Greco et al., 2007). In those with frequent abdominal pain (Scharff, 1997), as well as across children with diverse, chronic pain diagnoses (e.g., cystic fibrosis, juvenile chronic arthritis, diabetes melitus, osteogenesis imperfecta; Meijer et al., 2000; Palermo et al., 2014; Jones et al., 2021), endorsement of pain is associated with withdrawal from social activities. Generally, young people with chronic pain report a sense of loneliness and feelings of difference from their peers due to their pain experiences (Jones et al., 2021). Moreover, children who seek treatment for abdominal pain are more likely to experience physical discomfort, mental health concerns, and associated psychosocial impairment that persists into adolescence and adulthood (Campo et al., 2001). As such, timely and accessible psychological interventions are critical for youth with CAP.

While CAP is often not attributable to any organic disease (Di Lorenzo et al., 2005), one possible cause of CAP for some pediatric patients is median arcuate ligament syndrome (MALS). MALS is a vascular compression syndrome which occurs when the celiac artery is compressed by the diaphragm, leading to symptoms such as postprandial epigastric pain, nausea, and weight loss (Mak et al., 2013). The condition is rarely diagnosed, potentially due to the complexity associated with MALS being a diagnosis of exclusion, the poor understanding of the pathophysiology of pain associated with MALS, unpredictable treatment response, and lack of knowledge about the syndrome and its treatment (Skelly et al., 2018). Notably, while radiographic features of celiac artery compression are noted in upward of 50% of patients (Szilagyi et al., 1972), a much smaller percentage of individuals report the aforementioned symptoms or clinical profile associated with this diagnosis (e.g., 3.5%; Koç et al., 2018) and many report multiple barriers to diagnosis and treatment (Stiles-Shields et al., 2021), making both estimates of prevalence of MALS and clinical diagnosis challenging.

To receive a MALS diagnosis, patients must undergo a multipart diagnostic process involving radiologic studies and an extensive gastrointestinal workup (e.g., duplex ultrasound, computed tomography angiogram; Mak et al., 2013). Following this process and diagnosis, the MALS treatment team might recommend surgical release of the celiac artery. Surgery is voluntary and is therefore approached as a discussion with patients and their families, as the intervention does not guarantee full symptom relief (Skelly et al., 2018). Indeed, while the majority of patients report symptom relief and increased QOL following surgical intervention, about one third continue to experience CAP (Mak et al., 2013; Pather et al., 2020). Additionally, about half of pediatric patients who undergo surgery for MALS meet criteria for at least one psychiatric disorder, including depression and/or anxiety; these psychiatric symptoms do not appear to improve with surgical intervention (Mak et al., 2016; Stiles-Shields et al., 2018). Further, pre-surgical psychiatric diagnoses have been found to significantly predict lower post-surgical QOL for both pediatric and adult samples with MALS (Skelly et al., 2018; Stiles-Shields et al., 2018).

Due to the complexities and comorbidities of MALS, some teams have adopted an interdisciplinary assessment and treatment approach, consisting of a general pediatric surgeon, vascular surgeon, pain specialist, and psychologist (Stiles-Shields et al., 2018). This model is not universal across treatment settings. However, an interdisciplinary approach that includes a pre-surgical psychological evaluation has initial patient support as a beneficial practice (Stiles-Shields et al., 2021) and increases the likelihood of the identification of psychiatric comorbidities. While a pre-surgical psychological intervention is not always required to move forward with a MALS surgery, even when comorbidities are identified, it is possible that increasing the coping strategies of treatment-seeking pediatric patients with MALS may impact their post-surgical outcomes (Stiles-Shields et al., 2018). However, to date, there have been no psychological interventions designed specifically for this population.

For those individuals whose chronic pain persists post-surgically, effective coping strategies developed prior to surgery may be imperative for postoperative daily functioning. While not previously evaluated in pediatric MALS, it should be noted that in other pediatric populations undergoing surgery (e.g., osteosarcoma; Allen et al., 2020), openness to psychological interventions related to chronic post-surgical pain is associated with shorter length of pain treatment. Despite this, to date, the majority of interventions developed to prevent the development of pediatric post-surgical chronic pain have focused on hypnosis, which has proved “possibly efficacious” at best (Accardi and Milling, 2009). However, in adult populations, psychological interventions, including acceptance and commitment therapy (ACT; Weinrib et al., 2017) and cognitive behavioral therapy (CBT; Landry et al., 2020), are known to reduce use of opioid medication post-operatively, and diminish likelihood of onset of new pain. Such findings are promising, as they suggest that with developmentally appropriate adaptations, similar interventions which address pain-related cognitions may be useful in optimizing pain-related post-surgical outcomes in pediatric patients with MALS.

A likely intervention candidate for pediatric patients with MALS is CBT, which has been established as an evidence-based treatment for pain, including pediatric CAP, and has been found to simultaneously increase health-related QOL and decrease pain catastrophizing (Levy et al., 2010; Palermo et al., 2010; Thorn et al., 2011). In general, CBT for pain management consists of psychoeducation about the pain cycle, training in cognitive and behavioral strategies, and developing a long-term maintenance and relapse-prevention plan (Keefe, 1996). First, CBT for pain presents the cognitive triad, explaining that behavior and cognitions contribute to the experience of pain to emphasize the control the individual holds within their pain experience. Next, coping skills training occurs, which focuses on relaxation (e.g., progressive muscle relaxation, brief guided visualizations), cognitive restructuring (i.e., identifying and challenging catastrophic, pain-related cognitions), and activity management (i.e., activity pacing, pleasant activity scheduling). Finally, CBT for pain ensures the practice of implementing such strategies, as well as anticipating and problem solving for potential challenges and pain flares. To the best of our knowledge, no study has assessed the effectiveness of a psychological intervention in improving post-surgical outcomes for pediatric patients with MALS. Given that this population has similar psychosocial profiles to those with CAP (Sanders et al., 1994; Humphreys and Gevirtz, 2000; Robins et al., 2005; van der Veek et al., 2013), pediatric patients with MALS may also benefit from CBT.

Consideration of how CBT may be readily adapted and delivered for a disease population is crucial. Naturally, there may be treatment barriers when attempting to engage children with MALS in a pre-surgical psychological intervention. First, since MALS is a rare diagnosis, families often travel long distances to reach a MALS specialist (Stiles-Shields et al., 2021) additional and frequent travel for psychotherapy might be burdensome. Second, given the difficulties in reaching a diagnosis, patients and their parents may experience healthcare fatigue, which discourages them from further engaging with mental healthcare providers to receive psychotherapy. Third, patients with MALS experience chronic pain which impedes their daily functioning to varying degrees. Some patients find a task as simple as getting out of bed or taking a shower to be extremely difficult, whereas others might accomplish such tasks with limited interference (Stiles-Shields et al., 2021). Finally, motivation to engage with a psychological intervention may be limited based on patient- and parent-held beliefs about the nature of chronic pain. Given that the presenting pain is associated with a physiological anomaly (i.e., compression of the celiac artery), patients and their parents may ascribe to the biomedical model of pain. This emphasis on the biological aspects of disease may lead to a familial focus on signs and symptoms of the diagnosis, with attempts to alleviate distress primarily via correction of the underlying pathology (Craig and Weiss, 1990; Craig et al., 1996; Bendelow, 2013). As biomedical models favor pharmaceutical interventions, a view of MALS through this lens may discourage engagement with and beliefs about the utility of CBT as an effective component of treatment. However, as such models are known to neglect consideration of social determinants of pain, as well as the role of social learning in expression and experience of pain (Craig et al., 1996), interventions which provide psychoeducation surrounding the nature of pain and the pain cycle may serve to introduce patients to a more readily received transactional model of stress (Lazarus and Folkman, 1984; Craig et al., 1996), which teaches patients to better control their chronic pain by adapting their thoughts, judgments, and beliefs (Craig et al., 1996). In sum, a psychological intervention for pediatric patients with MALS would require a flexible approach, adapting to the barriers and needs of each patient. Offering remote delivery options for interventions (e.g., including telehealth or the use of digital platforms) may be one way to reduce the difficulty of accessing psychological services and overcome symptom-based (e.g., pain interference) and practical treatment barriers (e.g., distance to treatment site; Palermo et al., 2018). Further, an intervention would require psychoeducation and practical skills for the experience of daily pain.

Given the aforementioned barriers to treatment, as well as a lack of interventions specific to those with MALS, establishing the feasibility of a pre-operative CBT intervention for MALS is warranted. Determining feasibility for this pediatric disease population is crucial, given promising outcomes observed in the study of pre-operative psychological interventions in other populations with chronic pain. For example, children with chronic hip pain who received a pre-surgical, psychologically focused pain management intervention reported significantly lower postoperative pain intensity, as well as less pain distress and sleep disturbances (Berge et al., 2004). Additionally, other pre-surgical psychological interventions for elective surgical patients that included components of CBT for chronic pain (i.e., guided imagery) have demonstrated some support for improved psychological well-being and lower post-operative pain levels (Nelson et al., 2013). Such findings suggest that when CBT is adapted to address the needs and barriers of a disease population, pre-operative CBT may be an accessible, inexpensive and low-risk intervention through which psychosocial functioning may be scaffolded post-surgically. Given the dearth of literature on the feasibility of CBT in patients with MALS and the relative infrequency with which this diagnosis is made, a preliminary study which focuses on addressing said barriers and determining feasibility in a small sample is a crucial first step in determining the utility of pre-surgical CBT for MALS. Such a study allows for consideration of barriers to treatment (e.g., accessibility), allowing for adaptations and improvements to the delivery of treatment prior to engagement of a larger pilot or randomized controlled trial.

Pediatric patients with MALS present with chronic pain, lowered QOL, and frequently occurring comorbid psychiatric diagnoses, which may persist post-surgically. Therefore, the purpose of the current study was to: (1) implement a pre-operative CBT protocol with a focus on psychoeducation and coping strategies aimed to improve pain management, decrease subjective pain, and potentially reduce the need for surgical intervention; and (2) determine feasibility of a pre-surgical intervention for pediatric patients with MALS, with particular attention paid to adherence and patient election of whether to have surgery following intervention. Previous trials of CBT for pediatric chronic conditions have encountered enrollment refusal rates ranging from 0 to 75%; initial follow-up attrition ranges of 0–54%; and 0–59% for extended follow-up attrition rates (Karlson and Rapoff, 2009). We therefore hypothesized that feasibility would be established for pediatric patients with MALS based on the following metrics: (1) at least 50% consent to treatment when offered; (2) at least 85% of those who consent initiate treatment (i.e., complete at least one session); and (3) at least 75% complete treatment. While beyond the scope of a feasibility trial to assess impacts to clinical outcomes, we also aimed to characterize patient-reported symptoms across treatment (i.e., QOL, distress, pain, functioning, psychological characteristics).

## Materials and Methods

### Participants

Pediatric patients (<18 years of age) presenting to Comer Children’s Hospital, diagnosed with MALS, and who were eligible for surgical intervention based on an interdisciplinary assessment (Mak et al., 2013; Stiles-Shields et al., 2018) were invited to participate in a 7-week in-person or video-based pre-surgical CBT trial. Participants were excluded if they: (1) had difficulty reading, speaking, or understanding English; (2) were considered too medically unstable for outpatient psychotherapy; (3) were diagnosed with a psychotic disorder; (4) endorsed active suicidal or homicidal ideation; and/or (5) had a recent or planned change in antidepressant medication. A minimum age criterion was not enacted, as pediatric patients with MALS presenting for pre-surgical evaluation tend to be adolescent, as opposed to younger, pediatric patients.

### Procedure

In compliance with The University of Chicago Institutional Review Board, informed consent was obtained prior to the baseline assessment from the patient’s parent or guardian. Assent was obtained from all pediatric patients; any pediatric patients who turned 18 years old during the study period were re-consented as adults. Psychiatric comorbidities were assessed at baseline and post-surgery using a validated and structured clinical interview. Patient and parent-reported distress, pain interference and intensity, health-related QOL, and health status were assessed at four time points: baseline, following 4th session of CBT (mid-treatment), following 7th and final session of CBT (end of treatment), and 1 month after surgery. If patients and their families did not elect to undergo surgery, their 1-month post-surgical assessment was administered as soon as the decision was made and communicated with the research staff. Participants were compensated via gift card for study assessment completion. Specifically, they earned $25 for the baseline assessment (questionnaires and interview), $10 for the end of treatment/week 7 of CBT assessments (questionnaires only), and $25 for the post-surgery assessment (questionnaires and interview).

#### Pre-surgical Cognitive Behavioral Intervention

All participants underwent a seven-session, pre-surgical CBT protocol (see Table 1). Patients and their families could elect to engage in the intervention via in-person or video visits (conducted via a HIPPA-compliant Zoom account). Cognitive targets included identifying and challenging automatic thoughts related to pain, nausea, and stress, labeling and challenging cognitive distortions, and identifying and managing internalized and externalized stressors. Behavioral targets included coping/relaxation skills, pleasant activity scheduling, and time-based pacing. These targets were addressed across seven sessions. The intervention duration of seven sessions was selected to balance the need to address the core tenets of a CBT intervention for chronic pain with minimizing burden on patients (including their wait time to surgery). The intervention was administered by a postdoctoral fellow or master’s level psychology doctoral student, and was conducted with the pediatric patient alone, without a parent present. A licensed clinical psychologist supervised all treatment. All sessions were audiotaped and randomly selected for review by a clinical supervisor. Treatment fidelity was ensured utilizing the CBT scale for children and young people (CBTS-CYP; Stallard et al., 2014). Participation in the study was voluntary and did not affect the patient’s MALS care in any way. Recruitment was temporarily suspended mid-study (March 2020–September 2020) due to logistical challenges caused by the COVID-19 pandemic, including a temporary pause in funding.

**TABLE 1:** Session goals and strategies of pre-surgical CBT intervention.

**Session**	**Goals**	**Strategies**
Session 1: Psychoeducation about pain	Identify ways in which pain has impacted activities, thoughts, and feelings	Illustrate concepts using pain impact sheet; draw diagram showing cycle between pain, distress, and disability
Session 2: Progressive muscle relaxation and visual imagery	Introduce coping skills and relaxation techniques	Practice diaphragmatic breathing, PMR, and visual imagery in session
Session 3: Automatic thoughts and pain	Understand the relationship between thoughts, emotions, and pain	Introduce cognitive errors; Introduce ABC model
Session 4: Cognitive restructuring	Teach patient to challenge maladaptive thoughts about stress and pain	Use completed ABC worksheet to identify and challenge automatic thoughts related to pain; Use Thought Challenger worksheet
Session 5: Time-based pacing	Provide psychoeducation on importance of breaks and pacing techniques to prevent increased pain and later avoidance	Illustrate concept using Activity Pacing worksheet
Session 6: Pleasant activity scheduling	Understand the role of pain in activity withdrawal and low mood	Help patient identify and schedule activities he/she enjoys that are realistic and achievable
Session 7: Relapse prevention and flare-up planning	Normalize pain relapse; review progress	Discuss past pain relapses; collaboratively create plan for future relapses

*CBT, cognitive behavioral therapy; ABC, Activating Event, Beliefs, Consequences Model; PMR, progressive muscle relaxation. All sessions were conducted with the pediatric patient alone.*

#### Surgical Intervention

At the end of the 7-week intervention period, patients underwent an updated history, and physical and informed consent for surgical intervention was obtained. If surgery was still indicated and the family elected to proceed, minimally invasive (laparoscopic) surgical release of the median arcuate ligament was performed (please see Mak et al., 2013 for more details about the surgical intervention).

### Measures

All self-report assessments were administered and managed via the REDCap (Research Electronic Data Capture; Harris et al., 2009).

#### Interviews

##### Mini International Neuropsychiatric Interview for Children and Adolescents

The Mini International Neuropsychiatric Interview for Children and Adolescents (MINI-Kid) is a brief, structured diagnostic interview. Diagnoses are based on the Diagnostic and Statistical Manual of Mental Disorders, Fifth Edition (DSM-5) and International Statistical Classification of Diseases and Related Health Problems, Tenth Revision (ICD-10) criteria (Sheehan et al., 2010). The MINI-Kid was administered to pediatric participants by master’s level doctoral students and supervised by a licensed clinical psychologist.

##### Median Arcuate Ligament Syndrome Diagnostic Interview

The MALS Diagnostic Interview is a semi-structured interview designed for administration to pediatric patients and their families prior to and following surgical intervention for MALS (Anderson et al., 2011; Stiles-Shields et al., 2018). The MALS Diagnostic Interview was administered by master’s level doctoral students and supervised by a licensed clinical psychologist. Clinical information from this interview was used to supplement data from the MINI-Kid (i.e., provide parental/multi-informant input to diagnostic questions) to inform mental health diagnoses.

#### Self-Report Questionnaires

##### Demographic Characteristics

All pediatric participants were asked to report age, gender, current medical diagnoses, race/ethnicity, current living situation (e.g., with parents or independently), current work/school attendance, and socioeconomic status (SES). Demographic characteristics were administered at each time point.

##### Distress

The Kessler Psychological Distress Scale (K10) is a 10-item self-report questionnaire designed and validated to measure distress in youth and adults (Kessler et al., 2002, 2003). Scores range from 10 to 50, with scores categorized as non-clinical (<20), mild (20–24), moderate (25–29), and severe (30+). The K10 has previously been administered to pediatric and young adult patients with MALS (Stiles-Shields et al., 2021) and reliability was acceptable for the current pediatric sample at baseline (α = 0.86).

##### Quality of Life

The Child Health Questionnaire-87, child version (CHQ-CF87) is a self-report health status and QOL measure designed for children and adolescents, ages 5–18 (Landgraf and Abetz, 1997; HealthActCHQ, 2013). The CHQ-CF87 contains 12 concepts, including two single items: global health and change in health; and 10 multi-item scales: physical functioning; role/social limitations-emotional, behavioral, and physical; bodily pain/discomfort; behavior; global behavior; mental health; self-esteem; general health perceptions; family activities; and family cohesion. The CHQ-CF87 has previously been used with a small sample of pediatric patients with MALS (Joyce et al., 2014) and the measure has demonstrated acceptable to high reliability in school-based and clinical samples (attention-deficit/hyperactivity disorder; cystic fibrosis, end stage renal failure; HealthActCHQ, 2013). Authorization and a completed license to use the CHQ-CF87 was obtained for this study. Reliability for the CHQ-CF87 was acceptable at baseline for all concepts (αs = 0.76–92), with the exception of bodily pain/discomfort (α = 0.55). However, we have opted to still include the reporting of this two-item subscale to provide the characterization of this construct.

The Pediatric Quality of Life, version 4.0, child and teen reports (PedsQL) measures health-related QOL (Varni et al., 2001). The PedsQL yields a total score and subscale scores for physical, emotional, social, and school/work functioning. Higher scores indicate higher QOL, with a range of scores from 0 to 100. The PedsQL has been previously administered to pediatric and adult patients with MALS (Mak et al., 2013, 2016; Skelly et al., 2018; Stiles-Shields et al., 2018). The PedsQL child and parent versions were administered at all time points and demonstrated acceptable reliability at baseline (α = 0.88).

##### Pain

The PROMIS Pediatric Pain Intensity Form measures self-reported pain intensity as a one-item question for pediatric patients (Broderick et al., 2013). The PROMIS Pediatric Pain Interference Form measures the impact of pain on social, cognitive, emotional, physical, and recreational activities (Amtmann et al., 2010). The PROMIS Pediatric Pain Interference Form demonstrated acceptable reliability at baseline (α = 0.81). For both measures, higher scores indicate greater pain intensity or hindrance, respectively.

### Data Analysis

Descriptive analyses were used to characterize the sample, assess feasibility outcomes (i.e., attrition rates), and explore symptom-based outcomes across time (e.g., QOL).

## Results

### Participants

**FIGURE 1:**
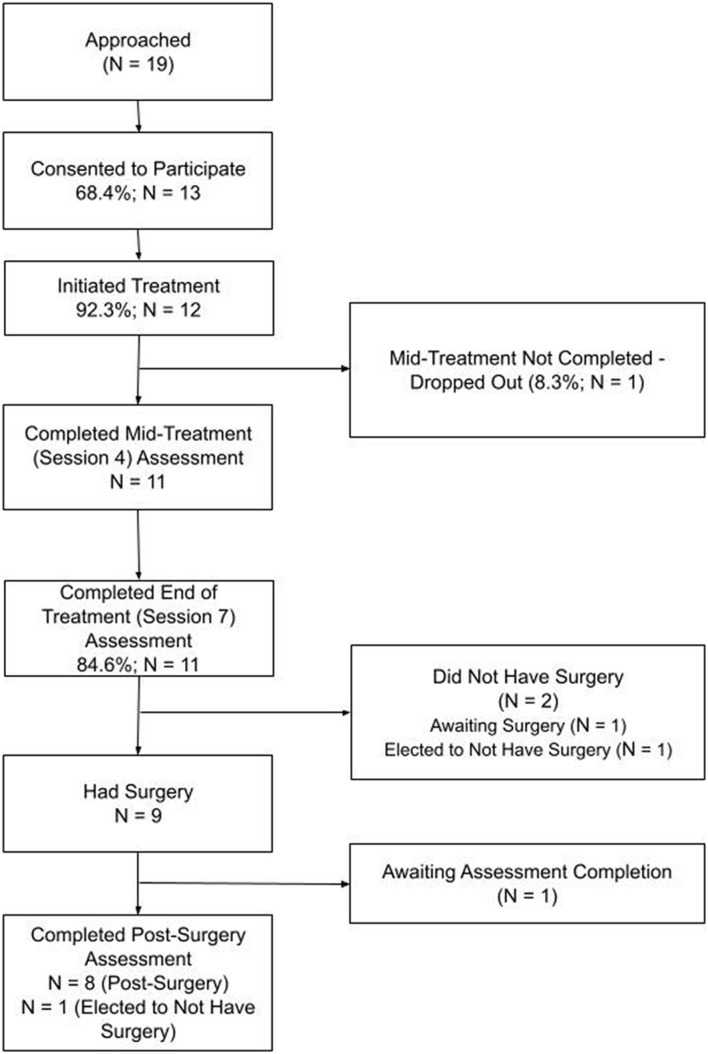
Consort chart for feasibility trial, assessment completion defined by parent and/or child participation.

The flow of participants through the feasibility trial is depicted in Figure 1 and sample characteristics are presented in Table 2. The sample for this study included 12 pediatric patients diagnosed with MALS, ages 13–17 (***M*** = 15.2 ± 1.7). The majority of the sample was female (91.7%), non-**H**ispanic/Latinx Caucasian (91.7%), and participated in treatment with their mothers (91.7%). The intervention trial began in August 2019, pausing due to the pandemic from March 2020 to September 2020. Two participants elected to complete the intervention face-to-face (16.7%), with the remainder opting for remote delivery.

**TABLE 2:** Sample characteristics, M (SD).

	**Baseline (*n* = 13)**	**Mid-treatment (*n* = 12)**	**End of treatment (*n* = 12)**	**Post-surgery follow-up (*n* = 10)**
Age	15.2 (1.72)	15.9 (1.46)	15.9 (1.60)	16 (1.4)
**Gender, *n* (%)**				
Female	12 (92.3%)	11 (91.7%)	11 (91.7%)	10 (100%)
Male	0	0	0	0
Non-binary	1 (7.7%)	1 (8.3%)	1 (11.1%)	0
**Racial, ethnic identity, *n* (%)**				
Non-Hispanic/Latinx Caucasian	12 (92.3%)	12 (100%)	12 (100%)	10 (100%)
Other (not specified)	1 (7.7%)	0	0	0
Highest level of education (grade)	9.1 (1.31)	–	–	–
Household income (US$)	$91,888 ($36,115.94)	–	–	–
**Parent participation, *n* (%)**				
Mother	11 (91.7%)	–	–	–
Father	1 (8.3%)	–	–	–
**CHQ-CF87**				
Global health	42.08 (26.24)	44.5 (24.55)	42.78 (29.49)	51.88 (25.63)
Physical functioning	69.75 (19.65)	65.19 (28.91)	55.97 (25.15)	61.57 (30.92)
Role/social limitations-emotional	68.52 (21.10)	58.89 (24.60)	58.02 (26.51)	76.39 (28.13)
Role/social limitations-behavioral	86.11 (19.03)	75.56 (34.27)	82.72 (24.91)	91.67 (16.53)
Role/social limitations-physical	74.07 (17.30)	61.11 (22.98)	59.26 (16.67)	72.22 (29.10)
Bodily pain/discomfort	30.83 (14.43)	21.0 (11.97)	22.22 (8.33)	40.0 (15.12)
Behavior	73.01 (18.21)	73.06 (11.98)	73.66 (14.10)	79.67 (13.64)
Global behavior	76.67 (20.26)	82.0 (16.53)	82.78 (14.39)	82.5 (15.35)
Mental health	57.29 (10.54)	52.03 (12.33)	52.43 (13.40)	63.09 (21.81)
Self esteem	63.54 (18.28)	64.29 (13.52)	62.30 (13.96)	70.54 (21.41)
General health perceptions	38.75 (17.90)	39.33 (17.02)	37.59 (15.25)	44.69 (19.13)
Change in health	2.25 (0.75)	2.2 (0.92)	2.22 (1.09)	3.75 (1.16)
Family activities	58.68 (20.91)	54.17 (20.31)	61.57 (16.37)	61.46 (19.38)
Family cohesion	3.92 (1.12)	3.66 (1.14)	4.16 (1.07)	4.05 (0.73)
**PedsQL**				
Physical	47.66 (14.0)	51.70 (20.56)	49.38 (15.92)	56.60 (19.73)
Emotional	51.57 (13.37)	49.09 (12.61)	49.5 (14.99)	60.0 (23.85)
Social	75.0 (24.03)	78.64 (21.34)	73.0 (21.11)	81.11 (18.16)
School	47.5 (12.34)	42.73 (16.49)	50.0 (16.67)	62.78 (30.43)
Total	54.44 (12.34)	55.04 (15.53)	54.67 (13.87)	64.0 (21.0)
K10	24.9 (6.02)	27.3 (6.63)	26.5 (6.08)	20.6 (8.03)
PROMIS pain intensity (0–10 scale)	6 (1.41)	5.8 (1.47)	6 (0.81)	4 (2.56)
PROMIS pain interference T-score	60.13 (5.41)	60.77 (4.93)	59.36 (6.41)	51.26 (9.88)

### Feasibility

Sixty-one patients presented to the interdisciplinary MALS team at Comer Children’s Hospital and The University of Chicago Medicine for a pre-surgical evaluation during the feasibility trial recruitment phases. Of these, 19 (31.1%) were under the age of 18 and met the criteria for a diagnosis of MALS. Six of these 19 pediatric patients and their families declined participation, expressing: (1) immediate disinterest in participation (*n* = 1; 16.7%); (2) delayed disinterest and/or lost to follow-up contact (*n* = 3; 50%); (3) disinterest due to being too busy (*n* = 1; 16.7%); or (4) disinterest due to seeking surgical intervention at a different location (*n* = 1; 16.7%). The other thirteen pediatric patients and their families consented to the study (68.4%). Nearly all consented participants initiated treatment (*n* = 12; 92.3%). Of these 12 participants, 11 completed the seven-session treatment, resulting in a retention rate of 91.7%. Only one participant dropped from treatment (8.3%), with the dropout occurring prior to the mid-treatment assessment. In terms of surgery, nine out of the 11 participants who completed treatment elected to undergo surgery (81.8%). Out of the two participants who did not have surgery, one is currently waiting for their surgery to be scheduled, while the other elected to not undergo surgical intervention; when the family communicated this decision, their post-surgery assessment was conducted.

### Patient Mental Health and Psychosocial Characteristics

#### Mental Health

Three participants did not meet criteria for any psychiatric diagnoses at the baseline interview. Nine participants met criteria for: depressive disorders [major depressive disorder (4), dysthymia (2)]; anxiety disorders [generalized anxiety disorder (3), separation anxiety disorder (2), social anxiety (2), obsessive compulsive disorder (1), agoraphobia (1), specific phobia (1), unspecified (1)]; adjustment disorders [with depressed mood (1)]; and attention-deficit/hyperactivity disorder [combined type (2)]. Average responses on the K10 across assessment time points indicated moderate (i.e., 25–30) to severe psychological distress (i.e., 30+).

#### Quality of Life and Pain

Table 2 displays the self-reported psychosocial functioning and pain scores across time. Health status and quality of life, as measured by the CHQ-CF87 and PedsQL, were similar at baseline to previous samples of pediatric patients with MALS (Joyce et al., 2014; Stiles-Shields et al., 2018). At baseline, participants rated their pain on average at a “6” (SD = 1.41) on a 0–10 scale on the PROMIS Pediatric Pain Intensity Form, while T-scores on the PROMIS Pediatric Pain Interference Form indicated primarily normative pain hindrance in engaging with social, cognitive, physical, and recreational activities.

#### COVID-19 Related Considerations

Study therapists informally queried patients during the final session regarding impacts of the COVID-19 pandemic on the efficacy of the intervention, session structure, and patient concerns and related symptom presentation. Patients endorsed increased concerns about health and safety, the need to adapt sessions related to time-based pacing and pleasant activity scheduling (due to changes in available resources and need for social distancing), diminished pain intrusion due to having fewer functional demands, and diminished daily structure.

## Discussion

The current study aimed to implement a pre-surgical CBT protocol emphasizing psychoeducation and pain coping strategies, determine feasibility of engaging pediatric patients with MALS in this intervention, and characterize the sample that engaged. Implementation began prior to the COVID-19 pandemic and included a remote delivery option, which promoted the ability to resume the intervention following a pandemic-related delay. Feasibility metrics were exceeded for engagement at the stages of consent (68.4% consented to treatment vs. goal of at least 50%), treatment initiation (92.3% completed at least one session vs. goal of at least 85%), and treatment completion (84.6% completed all sessions vs. goal of 75%). Additionally, nine participants (75%) elected to undergo MALS surgery following the CBT intervention. Of the patients and families who opted to participate in the intervention, pain experiences, QOL, and functioning were comparable to previous samples of pediatric patients with MALS (Joyce et al., 2014; Stiles-Shields et al., 2018). Further, mental health comorbidity was high, with 75% of the sample meeting criteria for at least one psychiatric diagnosis at baseline.

It was anticipated that multiple adaptations would be needed to effectively engage pediatric patients with MALS in a pre-surgical CBT intervention. Namely, a flexible approach in delivery options (face-to-face, video sessions) and emphases on pain psychoeducation and skill building around daily pain experiences and interferences. With the onset of the COVID-19 pandemic, remote methodologies became widely adopted and encouraged for clinical research efforts with pediatric populations (Stiles-Shields et al., 2020). However, at the onset of this feasibility trial, which predated the pandemic, additional efforts were required to justify to the Institutional Review Board (IRB) the need for a flexible delivery approach for pediatric patients experiencing CAP. We suspect that moving forward, many IRBs and institutions more broadly, will be open to remote delivery options. During treatment, multiple individual adaptations were made in response to patient-driven questions: (1) addressing symptoms related to comorbid conditions [e.g., Postural Orthostatic Tachycardia Syndrome (POTS)]; (2) means to improve medication adherence; and (3) how to plan for post-surgical adjustment (e.g., “How do I learn to eat and not be scared of food post-op?” “Will my hunger/fullness cues come back?”). While many of the questions were able to be easily woven into the established treatment, the questions regarding transition to post-surgical functioning support the likely benefit of offering supplemental post-surgical sessions.

Not surprisingly, the COVID-19 pandemic also appeared to impact the patient experience during the study period. First, multiple patients reported impacts to the health and safety of themselves and/or their family members (e.g., COVID-19 positive diagnoses). The pandemic may be viewed from a medical trauma perspective (Kazak et al., 2021), and as such, future adaptations of this protocol should consider these impacts regardless of the pandemic status (e.g., following a “return to normal”). Second, to comply with social distancing recommendations, time-based pacing and pleasant activity scheduling (sessions five and six) were particularly impacted. Some patients who participated in treatment early in the pandemic expressed initial feelings of relief that school attendance or social activities could be avoided, particularly during high pain days. As the pandemic continued, some patients reported that pain symptoms were less disruptive to their daily functioning, as they were not alone in missing typical activities (e.g., school, social outings). However, consistent with other youth populations during the pandemic (Brooks et al., 2020; Patrick et al., 2020), diminished daily activity structure relating to social distancing practices appeared to generally impact patients’ reported mood states and pain flare-ups. As such, study therapists became more creative in their collaborations with patients across sessions five and six to plan for in-home, near-by, and virtual activities that could provide structure, feelings of accomplishment/pleasure, and complied with social distancing recommendations. Considerations should be made following the pandemic to account for a potentially expansive view of positive activities that may be conducted virtually without interfering with the benefits of exposure to typical social activity experiences when in pain.

Pediatric samples with MALS commonly endorse comorbid psychiatric symptoms both pre- and post-surgically (Mak et al., 2016; Stiles-Shields et al., 2018). Further, depressive and anxious disorders are common in both pediatric CAP and chronic pain, more broadly (Youssef et al., 2008; Shelby et al., 2013; Fisher et al., 2018; Soltani et al., 2019). It was therefore anticipated that the sample would likely have a high prevalence of mental health diagnoses. Indeed, 67.7% met criteria for at least one DSM-V diagnosis at baseline. Establishing feasibility of engaging pediatric patients with MALS who are simultaneously experiencing comorbid psychiatric diagnoses is important, as the clinical team has reported a long-standing history of their recommendations for pre-surgical psychological interventions being met with resistance. Indeed, consistent with a focus on a biomedical model of pain (Craig and Weiss, 1990; Craig et al., 1996; Bendelow, 2013), the team reported that many pediatric patients with MALS and/or their parents would insist that surgical release of the celiac artery would improve the symptoms of MALS and subsequently, mental health would also improve. Similarly, others insisted that CBT would not be an effective intervention to address pain, as their pain was a result of an overt physiological cause and would therefore be unmodifiable through learning and applying coping skills. The current study therefore adds to a preliminary body of evidence that pediatric patients with MALS will engage in pre-surgical psychological interventions, and may report deriving long-term benefit from doing so (Stiles-Shields et al., 2021).

The current study had multiple strengths, including being the first implementation and evaluation of feasibility for a psychological intervention for pediatric patients with MALS. However, the findings should be interpreted in light of specific limitations. First, the sample was homogenous; it was comprised primarily of non-Hispanic/Latinx Caucasian females who presented with their mothers. While this is not unusual for samples of pediatric patients with MALS (Mak et al., 2013; Joyce et al., 2014; Stiles-Shields et al., 2018), consistency is not cause for complacency. Efforts are required to ensure better: (1) knowledge dissemination about pediatric MALS, (2) screening for MALS, particularly with marginalized populations with CAP, and (3) treatment access for pediatric patients with MALS, paying particular attention to systemic barriers which might be impeding the chance for underserved populations to receive a diagnosis and treatment given the resources needed to complete a diagnostic evaluation for MALS (Stiles-Shields et al., 2021). Second, to establish feasibility, only youth under the age of 18 were included. Consideration of developmental differences within the aforementioned age range is encouraged to readily evaluate how changes in cognitive development over these crucial years impact response to this intervention. Additionally, it is unclear how the current findings extend to older adolescent and young adult patients with MALS, many of whom presented for evaluation during the recruitment periods and expressed interest in a pre-surgical CBT intervention specific to MALS. Third, additional implementation outcomes and patient satisfaction with the intervention were not formally assessed. Engagement with treatment via treatment and assessment completion markers does not provide a full evaluation of feasibility or likelihood of clinical impact. Eliciting feedback from pediatric patients with MALS and their families, and including their voices in the development of a larger trial (e.g., stakeholder advisory board) is likely to promote better engagement and treatment experience. Fourth, this study evaluated feasibility of an intervention and was therefore not powered to evaluate clinical efficacy. Further, it is unclear how the effects of the COVID-19 pandemic may have contributed to patient-reported assessments and interviews. Given the impact of CBT-based interventions for other pediatric populations with CAP (Palermo et al., 2010; Groß and Warschburger, 2013; van der Veek et al., 2013), it is unlikely that CBT would have no positive effect for pediatric patients with MALS. However, the current study is unable to provide evidence for clinical impacts. Fifth, it must be acknowledged that the present study was underpowered to evaluate treatment efficacy, and findings related to feasibility should not be conflated with a statement of treatment efficacy. Evaluation of treatment efficacy is warranted in a larger, representative sample of pediatric patients with MALS. Finally, and as previously noted, this study was conducted during an international pandemic. Changes in how the medical system responded to the stress of this public health crisis delayed the testing of many patients suspected to have MALS (which is of note, as MALS is a diagnosis of exclusion, requiring multiple diagnostic tests). It is impossible to pull apart the effects of this phenomenon on the patients’ experience nor to determine whether the current sample differs from those who may have presented for evaluation under typical circumstances. Future research efforts should include: (1) evaluating the efficacy of pre-surgical CBT intervention on post-surgical outcomes with a larger sample and a longer follow-up (e.g., 6 months post-surgery); (2) evaluating the intervention across developmentally appropriate age groups [i.e., late childhood/early adolescence (9–13), mid-adolescence (14–17), late adolescence/emerging adulthood (18–25)]; and (3) incorporating more assessments of implementation and the generalizability of the intervention to other treatment sites.

The current study demonstrated initial feasibility for engaging pediatric patients with MALS in a pre-surgical CBT-based intervention. Despite a high prevalence of comorbid psychiatric diagnoses, a pandemic, and chronic pain symptoms experienced by patients with MALS, the intervention was deemed feasible based on protocol completion, suggesting that it would be beneficial to further evaluate the efficacy of the intervention with this population. Implementation of the intervention called for some adaptations specific for this population, including the ability to offer sessions both in-person and virtually, a focus on coping and skill building and psychoeducation, and flexibility to manage concerns about comorbid conditions and larger barriers related to the COVID-19 pandemic. Future research will include stakeholder input to conduct a pilot trial evaluating the efficacy of a pre-surgical CBT-based intervention for pediatric patients with MALS.

## Data Availability Statement

The raw data supporting the conclusions of this article will be made available by the authors, without undue reservation.

## Ethics Statement

The studies involving human participants were reviewed and approved by The University of Chicago Institutional Review Board. Written informed consent to participate in this study was provided by the participants’ legal guardian/next of kin.

## Author Contributions

CS-S, CS, and TD conceptualized and designed the study. All authors contributed to the drafting of the manuscript and approved the final version.

## Conflict of Interest

The authors declare that the research was conducted in the absence of any commercial or financial relationships that could be construed as a potential conflict of interest.

## Publisher’s Note

All claims expressed in this article are solely those of the authors and do not necessarily represent those of their affiliated organizations, or those of the publisher, the editors and the reviewers. Any product that may be evaluated in this article, or claim that may be made by its manufacturer, is not guaranteed or endorsed by the publisher.
